# Editorial: Interventional modalities for the prevention and management of childhood myopia

**DOI:** 10.3389/fopht.2026.1941158

**Published:** 2026-09-07

**Authors:** Rohit Saxena, Vinay Gupta, Meghal Gagrani, Jaspreet Sukhija

**Affiliations:** 1 Dr. R. P. Centre for Ophthalmic Sciences, All India Institute of Medical Sciences (AIIMS), New, Delhi, India; 2 Department of Ophthalmology, University of Pittsburgh Medical Centre, Pittsburgh, PA, United States; 3 Department of Ophthalmology, Post Graduate Institute of Medical Education and Research (PGIMER), Chandigarh, India

**Keywords:** childhood myopia, myopia, myopia control, myopia intervention, progressive myopia

Childhood myopia is no longer just a routine refractive error; it is increasingly being treated as a public health problem, with projections suggesting that by 2050 as many as half of the world’s population could be affected if current trends continue (1). The burden is especially visible in East and Southeast Asia, but rising prevalence is also being reported in low and middle income countries, where health systems are facing challenges. What once seemed like “simple” childhood myopia is now linked to later complications such as myopic macular degeneration, retinal detachment, glaucoma, and cataract, which makes early and structured control harder to ignore (2).

This Frontiers research topic focusses the spotlight on a very essential and practical question: how to prevent myopia onset and slow progression in real-world settings. Across all its articles, it covers epidemiologic risk modelling, digital screening and implementation science, pharmacologic and optical therapies, and opinions that push the field beyond refractive correction and toward active myopia management. The overall message is not that there is one answer, but that several complementary approaches need to work together across the course of a child’s visual development.

One stream of work in the Research Topic focuses on identifying which children are most likely to become fast progressors, using multivariable models that incorporate age, baseline refraction, parental myopia, near-work intensity, outdoor time and school environment. Prediction models developed in Asian cohorts show that simple clinical variables can stratify risk with reasonable accuracy, offering a basis for targeted surveillance rather than uniform annual refractions. Multiple articles emphasize that recognition should be early and systematic such as school-based screening, cycloplegic refraction and, where feasible, axial length measurement; so that interventional decisions are made before high myopia and structural damage emerge.

Another thread in the Research Topic explores how digital technologies and novel screening pathways can operationalize myopia control at scale. Stakeholder work around digital myopia screening programmes reveals both enthusiasm and caution: parents, teachers and clinicians appreciate the potential for reach, but raise concerns about data privacy, equity of access and integration with existing school health systems. These perspectives remind us that even the best interventional modalities may not deliver adequate results in practice if they are not embedded within socially acceptable, sustainable delivery models (3).

This Research Topic included review of pharmacologic and optical approaches, discussed considering current randomized trials and meta-analyses. Low-dose atropine, especially 0.01% and 0.05%, emerges as one of the main treatment options (4, 5), with evidence showing meaningful slowing of both refractive progression and axial elongation, even if results vary across ethnic groups and dosing schedules. The optical approaches covered include peripheral-defocus spectacle lenses such as DIMS designs, dual-focus soft contact lenses, and orthokeratology, all of which have shown clinically meaningful effects on slowing axial growth in controlled studies. Repeated low-level red-light therapy have shown effects on preventing axial elongation, though questions remain about long term safety and optimal treatment windows (6). Additionally novel spectacle designs with diffusion optics or contrast modulation shown promising short-term effect on axial length but their long-term efficacy and rebound effect, if any, yet to be accessed (7). The Topic also touches on combination therapy of pharmacologic plus optical intervention where additive/synergistic benefits appear in children who are inadequately controlled by monotherapy, reflecting a shift towards individualized, multi-modal regimens.

A distinctive feature of this Research Topic is consideration of primary prevention by intervening before myopia onset in high-risk, pre-myopic children. Opinion and perspective argue that delaying the onset of myopia by one or two years may translate into substantial lifetime reductions in axial length, potentially halving the risk of sight-threatening complications. At the same time, they also raise concern that prophylactic use of atropine or myopia-control lenses in children with no myopic refractive error raises ethical, adherence and cost concerns, and must be guided by robust risk stratification and careful counselling.

The Research Topic also highlights realities of clinical practice by emphasizing that discomfort, visual disturbances and behaviour can quickly erode the real-world impact of otherwise efficacious interventions. Reports on experience with peripheral defocus spectacle lenses, digital programmes and pharmacologic treatments shown that central acuity and trial outcomes are only part of the story; child and family acceptance, frame fit, centration, adaptation and day-to-day usability are equally critical to sustained success. Many authors call for structured troubleshooting and communication protocols, moving beyond ad-hoc advice to systematic support that keeps children engaged with their treatment over years rather than months.

Taken together, this Research Topic present childhood myopia care as a continuing management: it begins with behavioural and environmental measures, moves through risk-based screening and early recognition, and extends to tailored pharmacologic and optical treatment with regular follow-up. For clinicians, the takeaway is straightforward: myopia management can no longer stop at prescribing single-vision lenses. It calls for proactive, individualized care that is evidence-based but also realistic about local health-system constraints. For researchers and policymakers, the Topic highlights persistent gaps: data in non-isolated and syndromic myopia, long-term outcomes of emerging modalities, cost-effectiveness in low-resource settings, and scalable models that integrate digital tools, school health programmes and specialty clinics.

This Frontiers Research Topic shows that childhood myopia care now includes a wider range of interventions than it did even a few years ago (8), but having more options is only part of the story. Their value depends on whether they can be used in ways that fit local, clinical and public-health realities. As prevalence continues to rise, the task for clinicians, researchers, and public-health leaders is to turn these options into workable prevention and management strategies. The real goal is to ensure that fewer children progress to high myopia and fewer adults face avoidable sight-threatening complications later in life.
